# Static and Fatigue Properties of Rhenium-Alloyed Inconel 718 Produced by Powder Bed Fusion Additive Manufacturing

**DOI:** 10.3390/ma18030692

**Published:** 2025-02-05

**Authors:** Mariusz Frankiewicz, Michał Karoluk, Robert Dziedzic, Konrad Gruber, Wojciech Stopyra

**Affiliations:** Faculty of Mechanical Engineering, Wroclaw University of Science and Technology, Lukasiewicza 5, 55-371 Wrocław, Poland; michal.karoluk@pwr.edu.pl (M.K.); robert.dziedzic@pwr.edu.pl (R.D.); wojciech.stopyra@pwr.edu.pl (W.S.)

**Keywords:** laser powder bed fusion, Inconel 718, rhenium, microstructure, tensile properties, fatigue properties

## Abstract

Inconel 718 (In718) is the most widely used nickel-based alloy in additive manufacturing due to its favorable processability. However, In718’s high-temperature performance is not suited for the most demanding applications in the aerospace industry. Therefore, in this study, Inconel 718 powder was coated with 3% wt. rhenium (In718-Re) using AM’s in situ alloying capabilities to improve high-temperature properties. The proposed alloy’s mechanical performance was evaluated, focusing on the effects of post-process heat treatment and hot isostatic pressing following the laser-based powder bed fusion of metals (PBF-LB/M) processing. Static tensile tests conducted at room temperature and elevated temperatures (650 °C and 760 °C) demonstrated that the alloy has comparable strength to pure In718 according to ASTM F3055-14a—an ultimate tensile strength of 1247 MPa, yield strength of 909 MPa, and almost 2× higher elongation of 23.8%. Fatigue tests at room temperature indicated a fatigue limit below 400 MPa for 10^7^ cycles. Fractographic analysis revealed that fatigue performance was primarily impacted by a lack of fusion defects inherent to the PBF-LB/M process, highlighting the need for optimized powder preparation and processing parameters to minimize defect formation. While rhenium addition shows limited benefits in Inconel 718, this study underscores the potential of in situ alloying through powder surface modification as a flexible method for incorporating high-melting-point elements into nickel-based alloys for tailored alloy design in additive manufacturing.

## 1. Introduction

Additive manufacturing (AM) offers an approach to flexible component fabrication with complex geometrical structures and mechanical properties tailored for selected application areas. It brings new opportunities for extending traditional material science and creating new materials by allowing the modification of the composition and material structure of the manufactured component through in situ alloying [1]. AM processes allow the use of new design methods through generative design and topological optimization approaches, enabling the reduction in the weight of manufactured components while maintaining their mechanical properties [2,3]. Among the various AM techniques, the powder bed fusion (PBF), with a 54% market share, is the most prominent in the field of metal AM [4]. Major representative technologies, laser-based powder bed fusion of metals (PBF-LB/M) and electron-based powder bed fusion of metals (PBF-EB/M), stand out for effectively producing high-strength components and for the ability to significantly reduce the amount of raw materials consumed compared to conventional manufacturing techniques [5,6].

On the other hand, AM components usually exhibit inhomogeneous structures and defects, such as porosity, microsegregation, and high levels of residual stresses in as-built conditions [7]. The additional post-treatment helps to reduce the anisotropic structure of the AM-processed materials and minimize the influence on the mechanical properties of the final objects [8].

AM can be an alternative solution to conventional machining technologies when machining difficult-to-process materials with high hardness and low thermal conductivity, such as nickel-based Inconel alloys. It eliminates the disadvantages of machining and casting processes, such as loss of material turned into chips, low removal rates, extended lead times, and high scrap rates due to environmental issues [7].

Inconel 718 (In718), a Ni–Fe–Cr alloy modified with niobium, is renowned for its high strength, resistance to creep, and excellent fatigue properties at elevated temperatures up to 700 °C. The alloy’s high strength is primarily due to the precipitation of γ′ (Ni_3_(Al, Ti, Nb)) and primarily γ″ (Ni_3_Nb) phases within its face-centered cubic (fcc) matrix. These precipitates significantly enhance the mechanical properties, including yield and tensile strengths, which are maintained even at elevated temperatures [4]. These attributes make In718 a preferred material for manufacturing the high-temperature working components of gas turbines, aircraft engines, and turbocharger rotors [9,10] and in the nuclear industry [11]. Additionally, this alloy type slowly forms precipitates due to its good weldability, minimizing the risk of hot cracking during welding. However, the alloy’s high hardness and low thermal conductivity present challenges for conventional machining and forming processes, particularly for complex components [10,12]. Due to the technological issues encountered during the processing of this material using subtractive manufacturing technologies, interest in alternative methods, such as additive manufacturing, is growing [2,7]. Due to the presented characteristics, In718 was among the first materials studied for processing using AM [13], and, to date, it remains the most used nickel-based alloy in additive manufacturing.

The mechanical properties of In718 processed by AM can be enhanced through modifications of its composition with alloying addition [14]. In718 has been modified with in situ additives like WC-W_2_C [15], CoAl_2_O_4_ [16], and MC-type carbides [14], achieving substantial grain refinement after heat treatment due to the Zener pinning effect. This refinement enhances the tensile strength and creep resistance. Alloying with W decreases the interdendritic region and refines the grain size in In718, which can improve the corrosion resistance of Inconel 718-W. However, increased W addition can enhance the galvanic microcorrosion due to the presence of precipitated Laves and Fe_2_W phases [17]. Alloying with Ti increases the fraction of the γ′ phase and improves the abrasive wear resistance of the modified Inconel 718 [18]. In718 was also alloyed with rhenium to improve high-temperature strength and creep resistance, allowing its use in more demanding applications [11]. The addition of rhenium aims to exploit its positive effects on the microstructure and mechanical performance of the alloy, particularly under high-stress and high-temperature conditions. Research on additive manufacturing of rhenium with high-temperature-resistant materials, such as tungsten, indicates its potential to improve mechanical properties, such as high-temperature hardness, and to reduce technological issues by minimizing cracking [7,19].

This study examines the influence of rhenium alloying and post-process heat treatments, including hot isostatic pressing (HIP), on the material structure of PBF-processed IN718-Re. Tensile strength and fatigue performance were evaluated to guide the optimization of high-temperature and high-stress capabilities in additively manufactured In718-Re components designed for aerospace and other demanding applications [8].

## 2. Materials and Methods

### 2.1. Powder Preparation

**Figure 1:**
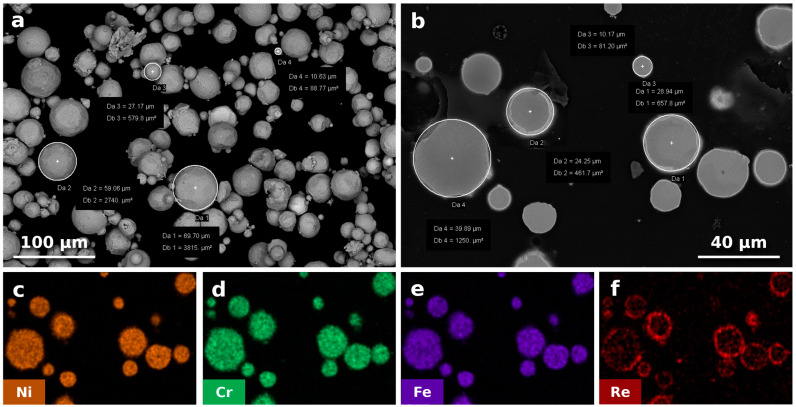
In718 powder with surface modified by Re (In718-Re) used in this research. (**a**) Powder morphology observed using SEM in BSE mode. (**b**) Rhenium distribution on the surfaces of powder particles, powder cross-section mounted in resin, SEM, BSE. (**c**–**f**) EDS maps showing the distribution of the following alloying elements: (**c**) Ni, (**d**) Cr, (**e**) Fe, and (**f**) Re, based on Figure 1b.

The In718 powder used in this research has been surface-modified by rhenium using a thermal reduction process (patent pending P.443143) [20] by the Lukasiewicz Institute of Non-Ferrous Metals in Gliwice (Poland). Figure 1 shows the powder morphology along with EDS maps of the major alloying elements and rhenium. The amount of rhenium addition was 3% by weight, determined based on the results of previous work [11].

### 2.2. Sample Fabrication

Inconel 718 powder with its surface modified by rhenium was used as a feedstock to manufacture specimens. The specimens were fabricated using a laser-based powder bed fusion of metals (PBF-LB/M as defined in the ISO/ASTM 52900 [21] and ISO/ASTM 52911 [22] standards) process. An SLM 250 system (Realizer, Germany) equipped with a 100 W fiber laser was used. The process parameters used were as follows: 50 µm layer thickness, 1200 µs scanning point time, 60 µm scanning point distance, and 160 µm hatch distance. The build platform was preheated to 250 °C prior to the process, and this temperature was maintained throughout the entire duration of the procedure. The samples were fabricated with an inclination angle of 45° between their symmetry axis and the build platform plane. The process was conducted in a technical argon protective atmosphere.

Table 1 outlines the experimental conditions and standards applied in the study. The experimental campaign includes four series of near-net shape specimens manufactured for static tensile strength tests at room and elevated temperatures and fatigue tensile tests. The specimens produced via PBF-LB/M were post-processed by machining according to ASTM E8/E8M-22 [23] for static tensile strength tests and ASTM E466 [24] for fatigue tests.

### 2.3. Heat Treatment and Hot Isostatic Pressing Post-Treatment

Prior to testing, a post-treatment process was employed to homogenize the structure and improve the mechanical properties of the In718-Re samples. The HT process consisted of three steps: hot isostatic pressing (HIP) and solution annealing + double aging (HT). HIP was performed using an EPSI 300-125/200 hot isostatic press (EPSI NV, Temse, Belgium), while the remaining heat treatment steps were conducted in a laboratory furnace (Czylok, Poland).

First, HIP was applied to reduce post-AM defects, with samples subjected to 1185 °C under 100 MPa pressure for 4 h. Heating and cooling rates were maintained at 600 °C per hour. The HIP temperature of 1185 °C was selected based on the ASTM F3055-14a standard [25]. Choosing a temperature in the higher range was intended to enhance diffusion kinetics, facilitate porosity healing, and promote the effective homogenization of rhenium within the alloy.

After HIP, the samples underwent solution annealing at 1100 °C in an argon atmosphere for 1 h, followed by rapid water cooling to retain the high-temperature microstructure and prevent precipitation of undesirable phases, such as σ or Laves. The heating rate for this stage was 200 °C per hour.

A two-stage artificial aging process followed to precipitate strengthening phases: 8 h at 718 °C for γ″ phase formation and 10 h at 621 °C for γ′ phase formation, both in an argon protective atmosphere with a heating rate of 200 °C per hour. After aging, the samples were allowed to cool naturally in air.

Previous studies have effectively utilized similar solution annealing and artificial aging parameters, as detailed in [26].

### 2.4. Material Characterization

The microstructure of the cross-section of the as-built and HIP-treated samples was observed using SEM Zeiss Sigma 500VP (Zeiss, Oberkochen, Germany) equipped with EDS and EBSD detectors. The samples were sectioned using metallographic cutting in a plane aligned with the build direction. Subsequently, the samples were hot-mounted in epoxy resin. The mounted metallographic specimens were ground using abrasive papers from #80 to #2000 and mechanically polished with a colloidal silica polishing agent. For microstructural observations, the specimens were etched with Gliceregia reagent (3 parts HCl, 2 parts glycerin, and 1 part nitric acid) for approximately 30 s. Chemical composition data were acquired at an accelerating voltage of 20 kV, with a working distance of 9 mm for BSE imaging and 13.7 mm for EBSD measurements. The EBSD specimens underwent mechanical polishing for 2 h. EBSD patterns were collected using an EDAX Octane camera (EDAX, Pleasanton, CA, USA), and measurements and analyses were performed with a fully automated TEAM™ EBSD analysis system integrated with the Zeiss Sigma 500VP scanning electron microscope.

The porosity evaluation was conducted on a single sample at two stages: in the as-built state and after HIP treatment. The X-ray computed tomography (XCT) Zeiss Metrotom 1500 (Zeiss, Oberkochen, Germany) system was used. The resolution of data for the In718-Re specimens was 20 μm at a tube voltage of 120 kV and a current of 80 μA.

### 2.5. Tensile Test

Tensile tests at room temperature were carried out according to the ASTM E8/E8M-22 standard [23] using an Instron 3384 (Instron, Norwood, MA, USA) tensile testing machine equipped with a 10 kN load cell with an accuracy class of 0.5% and a non-contact video strain gauge (AVE 2663-821). The tests were conducted with a crosshead speed of 2 mm/min and continued until sample failure. Three measurements were performed for each series.

Tensile tests at elevated temperatures of 650 °C and 750 °C were carried out using a Zwick Roell 1478 (Zwick/Roell, Ulm, Germany) tensile testing machine equipped with a 100 kN load cell and a heating chamber of a 3-zone furnace. Elongation was measured using a contact extensometer equipped with ceramic rods. The tests were conducted with a crosshead speed of 0.9 mm/min and continued until sample failure. Three measurements were performed for each series.

Hardness measurements were performed using a Zwick/Roell ZHVμ-A (Zwick/Roell, Ulm, Germany) Vickers hardness tester at the load of 0.3 kgf (HV0.3).

### 2.6. Fatigue Test

The fatigue tests were carried out according to the ASTM E466 standard [24] using an Instron 8872 (Instron, Norwood, MA, USA) servo-hydraulic testing machine equipped with a 25 kN load cell. The specimens were tested with a tension–tension sinusoidal loading cycle, maintaining a constant stress ratio (maximum stress/minimum stress) of R = 0.1 and a frequency of 50 Hz.

For the measurement series, tests were performed at three different stress levels, with three measurements taken at each level to determine the effect of stress (σ) on the number of cycles to failure. The results were plotted on a stress vs. cycles to failure (S–N) curve. The fatigue tests were conducted up to 10^7^ cycles (run-out limit).

## 3. Results

### 3.1. Microstructure

**Figure 2:**
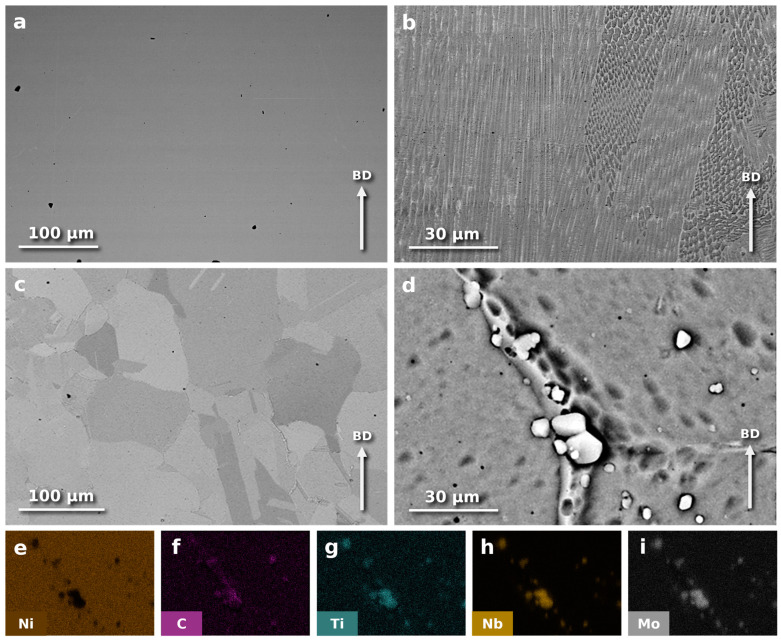
Microstructure of PBF-LB/M-processed In718-Re alloy. (**a**) As-built, not-etched, observed using SEM, BSE. (**b**) As-built, showing low-angle boundaries between columnar subgrains, with the interior of grains revealing dendritic microsegregation of alloy components; etched, SEM, BSE. (**c**) In718-Re alloy after HIP + HT, featuring large grains of the γ phase with a significant number of twin boundaries; etched, SEM, BSE. (**d**) In718-Re alloy after HIP + HT, magnified view of the junction of three grains, showing visible precipitates of Cr and Nb carbides; etched, SEM, BSE. (**e**–**i**) EDS maps based on Figure 2d, showing the distribution of the following alloying elements: (**e**) Ni, (**f**) C, (**g**) Ti, (**h**) Nb, and (**i**) Mo.

The representative as-built microstructure of the PBF-LB/M-fabricated In718-Re in a polished, non-etched state has been presented in Figure 2a. It shows a relatively large surface area of the sample (width of 3 mm) prepared along the build direction of the sample (z-direction). Typically, in situ alloying in powder bed fusion is performed using feedstock powder blends [27]. This powder preparation method often results in unmelted alloying particles within the alloy matrix due to conglomeration from inadequate powder blending [28] or the accumulation of high-melting-point particles at the solidification front of the melt pool [29].

The micrograph in Figure 2a was obtained using a BSE detector to identify the rhenium-rich zones that had not melted or partially melted. Due to the significant difference in density between rhenium (21.02 g/cm^3^) and Inconel (approximately 8.19 g/cm^3^), regions with a higher concentration of rhenium appear brighter in the BSE detector images (as presented earlier in Figure 1b). The observations did not reveal any bright areas, as observed in previous studies carried out by the authors on processing In718 and Re powder mixes [11]. This indicates the effective dissolution of rhenium in PBF-LB-processed In718-Re. In addition, the non-etched micrograph shows no cracks or other significant internal defects, excluding a few larger keyhole pores and smaller gas pores (most likely filled with trapped argon) of no more than 2 µm in size. After the additional heat treatment, no regrowth of pores, known as thermal-induced porosity (TIP) [30], was observed.

The porosity measurements performed by XCT on a sample in the as-built state and after the HIP process presented values of 0.028% and 0.021%, respectively. The majority of pores were located in a subsurface area of a sample.

The microstructure of the etched, as-built In718-Re sample is shown in Figure 2b. Similar to the non-etched sample, the structure does not exhibit cracks, inclusions, or unmelted (partially melted) rhenium particles. The structure is typical for laser-processed, single-phase nickel-based alloys and consists of columnar γ-phase grains aligned with the build direction. These grains grow along the direction of the highest thermal gradient, which is usually perpendicular to the powder bed surface. Parallel dendritic cells within columnar γ-phase grains are observed within columnar grains. These cells form packets with small misorientation angles (2° < θ < 5°) separated by low-angle grain boundaries (5° < θ < 15°) within larger grains [26], generating a high-density dislocation network [8]. Evidence of layer-by-layer manufacturing can also be observed in the form of horizontal laser melting traces due to local microsegregation.

The microstructure of an In718-Re alloy sample after HIP and heat treatment is shown in Figure 2c,d. It consists of large γ-phase grains with numerous twin boundaries (Figure 2c). No evidence of a retained as-built directional structure was observed. Figure 2d provides a magnified view of the boundaries of three grains, highlighting visible carbide precipitates. At grain boundaries, (Nb, Ti, Mo)C carbides and traces of an Nb-, Ti-, and Mo-rich liquid film are observed, as confirmed by the EDS maps in Figure 2e–i. The presence of the liquid film is characteristic of the heat-treated high-temperature In718 alloy processed by PBF-LB/M and subsequently HIP, resulting from local liquid film formation during rapid heating above the pseudo-ternary eutectic temperature and its solidification upon cooling. These features, including the liquid film and (Nb, Ti, Mo)C carbides, are typical of the high-temperature treatment applied in this study [26].

**Figure 3:**
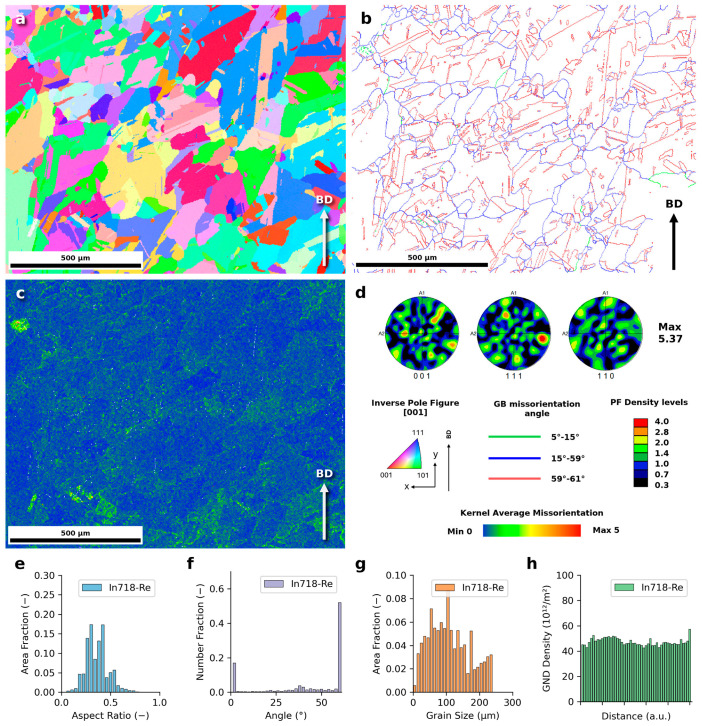
Results of EBSD investigation of In718-Re after HIP and HT: (**a**) Inverse pole figure (IPF) map with coloring in the XY plane. (**b**) Grain boundary map with twin boundaries marked in red. (**c**) Kernel Average Misorientation (KAM) map. (**d**) Pole Figures (PF) and legend. Histograms of grain aspect ratio by area fraction (**e**), misorientation angle (**f**), grain size distribution by area fraction (**g**), and GND density from left to right of the map (**h**).

The EBSD analysis results for an In718-Re alloy sample after hot isostatic pressing and further heat treatment in the building direction plane are presented in Figure 3. The combined effects of high pressure, elevated temperature, and extended holding times during HIP lead to significant grain growth and homogenization in the alloy microstructure (Figure 3a). This process is strongly associated with annealing twins (Figure 3b), previously identified as a primary mechanism driving the recrystallization of powder bed fusion-processed In718 alloys [26]. Twin boundaries, defined by a 60° misorientation, account for approximately 50% of all grain boundaries (Figure 3f). The average aspect ratio of grains is 0.36 (Figure 3e), and the average grain size, calculated by area fraction, is 111 µm, which aligns closely with the values observed for pure Inconel 718 after similar post-treatment [14]. The significant grain growth and twinning observed in the post-treated In718-Re alloy are expected to enhance ductility and reduce residual stresses, contributing to the improved mechanical stability, as discussed later.

Despite the homogenization and near-equiaxed grain morphology, the In718-Re alloy retains some residual texture originating from the PBF-LB/M process. The peak pole density is measured at 5.37, and the pole figures (Figure 3d) show a semi-random texture with remnants of Goss texture, characteristic of as-built In718 [14]. This suggests that, while the post-treatment significantly reduces texture anisotropy, traces of epitaxial grain growth from the original PBF-LB/M microstructure persist (Figure 2b). The residual Goss texture and epitaxial growth remnants may introduce a degree of anisotropy, potentially influencing the mechanical performance under directional loading.

The kernel average misorientation (KAM) map (Figure 3c) and the geometrically necessary dislocation (GND) density map (Figure 3h) further illustrate the microstructural changes. The average GND density is calculated as 35 10^12^/m^2^, consistent with values reported for pure In718 subjected to HIP, solution annealing, and aging [14]. As the previous studies showed, the as-built In718 alloy can exhibit GND densities that are an order of magnitude higher. The uniform KAM values and relatively low GND density confirm the recrystallized and homogenized state of the In718-Re alloy after the complete post-treatment process.

The high fraction of twin boundaries could positively impact creep resistance, as such boundaries are effective barriers to dislocation motion. However, while the observed KAM and GND values, indicating low dislocation density, suggest potential fatigue resistance improvements, other factors may offset this benefit. For instance, the presence of twin boundaries and internal defects could counteract the positive effects of reduced stress on fatigue performance.

### 3.2. Tensile Strength

**Figure 4:**
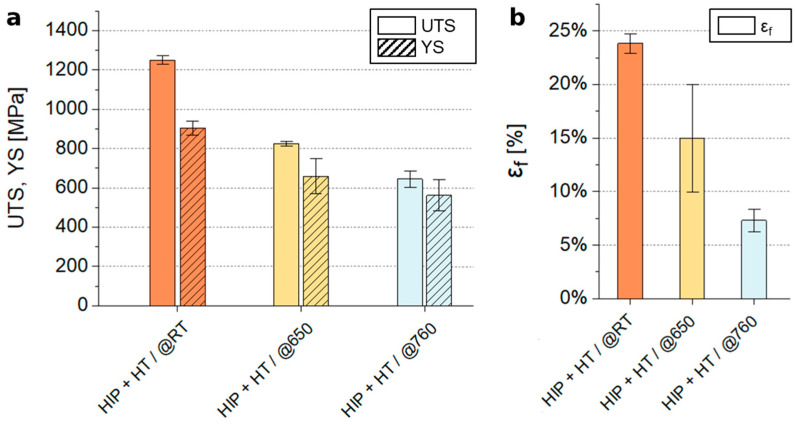
Tensile properties of In718-Re samples at various temperatures: room temperature, 650 °C, and 760 °C. (**a**) Ultimate tensile strength (UTS) and yield strength (YS). (**b**) Elongation (ε_f_). Error bars represent 95% confidence intervals (α = 0.05).

Figure 4 presents the analysis of tensile test results for Inconel 718 samples. According to the ASTM F3055-14a standard [25], additively manufactured In718 alloy subjected to HIP, solution annealing, and aging is required to meet minimum tensile properties, including an ultimate tensile strength (UTS) of 1240 MPa, yield strength (YS) of 920 MPa, and elongation of 12%. In718 alloyed with 3% Re (HIP + HT/@RT) exhibits a comparable UTS of 1247 MPa and a slightly lower YS of 909 MPa but demonstrates a significantly higher elongation of 23.82%.

Additionally, the HV0.3 hardness measurements were conducted in the build direction for IN718-Re samples in the as-built, HT, and HIP + HT conditions. The resultant values were 301 ± 8, 493 ± 34, and 460 ± 14, respectively.

At 650 °C, the HIP + HT/@650 series shows a further reduction in the mechanical properties, with UTS decreasing to approximately 826 MPa, YS to about 661 MPa, and elongation dropping to around 15%. This trend persists in the HIP + HT/@760 series, where UTS declines to approximately 645 MPa, YS to around 565 MPa, and elongation significantly decreases to about 7.3%.

**Figure 5:**
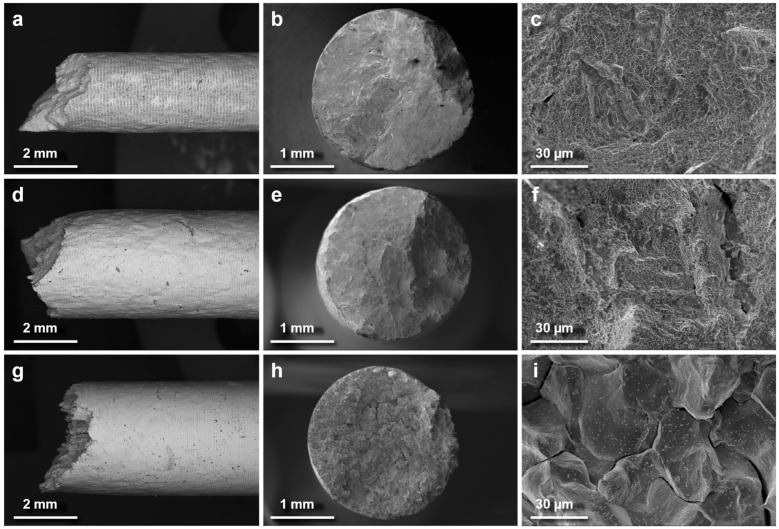
Fractures in the In718-Re HIP + HT specimens observed at various temperatures (RT, 650 °C, 760 °C). SEM micrographs of fracture surfaces: (**a**–**c**) HIP + HT/@RT, (**d**–**f**) HIP + HT/@650, (**g**–**i**) HIP + HT/@760.

An examination of the fracture surfaces of HIP + HT/@RT (Figure 5a–c) provides further insight into the material’s behavior. The fracture occurred at a 45° angle, perpendicular to the build direction (Figure 5a). Despite the considerable elongation of 22%, no necking was observed. The fracture surface displayed a well-developed morphology with numerous dimples of varying sizes and shapes, indicating ductile fracture (Figure 5b). Moreover, a deformation relief on the lateral surface of the sample is visible (Figure 5a). This, combined with the dimpled fracture surface and high elongation, suggests a homogeneous stress distribution [15], likely due to the homogenized structure of the alloy after the applied heat treatment.

The fracture surface in the HIP + HT/@650 series (Figure 5d–f) showed a developed morphology with numerous dimples of varying sizes and shapes (Figure 5f), similar to the HIP + HT/@RT series, indicating ductile fracture. This suggests that even at 650 °C, the material could undergo plastic deformation before failure. However, in the HIP + HT/@760 series, the fracture surface (Figure 5h) displayed a quasi-cleavage character, as shown in Figure 5i. Cleavage ridges and surrounding facets were observed, with the ridges exhibiting relatively sharp and elongated edges. This indicates some plastic deformation during their formation. The transition to a quasi-cleavage fracture mode at 760 °C indicates reduced ductility, with the material exhibiting increased brittleness at elevated temperatures, suggesting a usability limit below the test temperature.

### 3.3. Fatigue Strength

**Figure 6:**
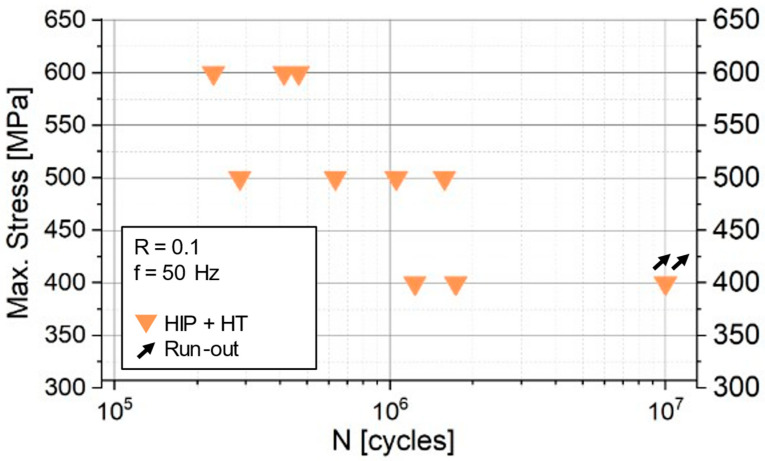
S–N plot of fatigue test results: the relationship between the applied stress and the number of cycles to failure of the In718-Re/HIP + HT specimens.

Figure 6 presents the results plotted on an S–N diagram, showing the correlation between applied stress and the number of cycles to failure for the tested specimens. The fatigue strength, representing the stress level at which the specimens endure 10^7^ cycles without failure (run-out stress), is found to be below 400 MPa.

**Figure 7:**
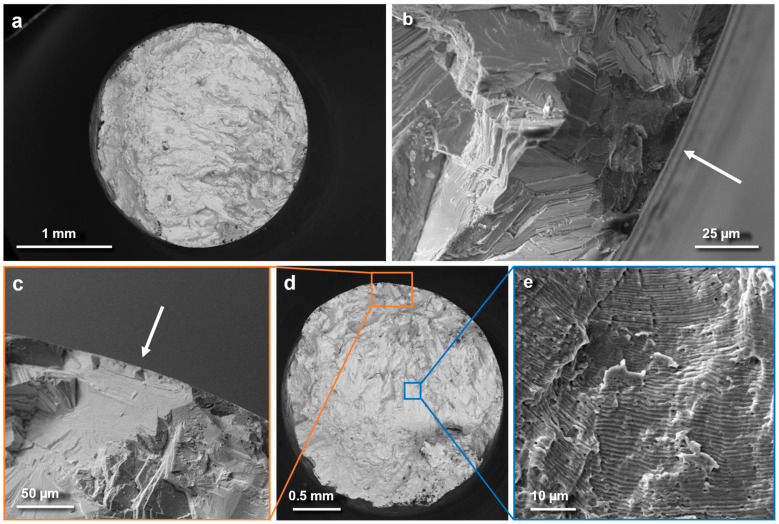
Fracture surfaces of samples subjected to fatigue tensile testing at room temperature: (**a**) Overall view of the fracture surface at 400 MPa. (**b**) Magnified detail of a defect observed within the fracture surface at 400 MPa. (**c**) Magnified detail of a defect on the fracture surface at 600 MPa. (**d**) The overall view of the fracture surface at 600 MPa. (**e**) Fatigue striations visible within the fracture surface at 600 MPa. White arrows indicate crack initiation sites.

A microscopic analysis of the fracture surfaces of fatigue samples is presented in Figure 7. The fractures show that fatigue cracks are initiated due to two different mechanisms depending on the stress level. At stress levels of 400 MPa, 500 MPa, and 600 MPa, cracks were predominantly initiated at internal defects located near the specimens’ surface. These lack-of-fusion (LOF) defects, characteristic of the PBF-LB/M process, remain in subsurface areas despite the application of HIP treatment, which is intended to eliminate such defects. This is evident from the presence of a flat LOF defect in the fracture area (Figure 7b, white arrow). Such pores were also observed in a subsurface region during XCT control measurement of the HIP-ed sample. Such defects were not observed in the cuboid samples, as shown in Figure 2a, possibly due to differences in sample geometry, which could influence defect formation during the PBF process. A properly optimized HIP process can reduce the porosity of an In718 alloy from levels as high as 7–8% to as low as 0.01% [31]. Nevertheless, as demonstrated in the referenced study [31], high temperature and pressure do not always result in optimal densification. Moreover, lack-of-fusion (LOF) pores, especially those located near the surface or connected to it, cannot be completely eliminated using HIP alone [32]. In the In718-Re samples, the HIP process effectively reduced internal porosity within the bulk material. However, flat, subsurface lack-of-fusion (LOF) defects persisted in the structure, acting as critical initiation sites for the fatigue failure mechanism.

At a stress level of 600 MPa, an additional initiation mechanism was observed. Fatigue cracks in these samples originated from twin boundaries (Figure 7c, white arrow). These boundaries, visible as flat regions or facets on the fatigue fracture surfaces, are a significant feature of the material’s microstructure. The twin boundaries in the analyzed alloys are substantial, exceeding 100 μm in size, as confirmed by Figure 3. According to the literature, twin boundaries are potential crack initiation sites [33]. This suggests that while internal defects near the surface dominate crack initiation at lower stress levels, twin boundaries become a critical factor at higher stress levels, specifically at ≥600 MPa.

Overall, the material demonstrated consistent fatigue behavior across different stress levels, with internal defects near the surface playing a key role in crack initiation at all stress levels and twin boundaries contributing significantly at higher stresses. Despite the HIP treatment improving the material’s density, these sites remained critical for crack growth. The fatigue nature of the failure is further supported by visible striations on the fracture surfaces, as shown in Figure 7e, which illustrates the progressive advancement of the crack during cyclic loading.

## 4. Discussion

The results presented in this study demonstrate that processing Inconel 718 using the PBF-LB/M method with surface-modified powder containing 3 wt.% rhenium resulted in the complete dissolution of rhenium particles and facilitated the in situ alloying of Inconel 718 with rhenium. The absence of unmelted rhenium particles in the as-built microstructure indicates that the thermal reduction process used for powder preparation was highly effective. Unlike traditional powder blends, the coated powder approach resulted in a homogeneous distribution of rhenium, thereby eliminating issues related to segregation or incomplete alloying due to unmelted additive particles.

Additional thermo-mechanical processes (HIP + heat treatment) were applied to the fabricated samples to eliminate the as-built structure and heal the internal defects typical of the PBF-LB/M process. These processes contributed to the observed improvements in ductility. Despite the application of HIP, certain near-surface defects remain, highlighting the limitations of HIP processing. While the defect size and distribution density are reduced, these defects continue to act as potential crack initiation points under cyclic loading, as demonstrated by fatigue testing. The addition of rhenium did not affect the alloy microstructure before or after HIP and after heat treatment. The rhenium addition was dissolved in the matrix as a solid solution, potentially contributing to solid-solution strengthening effects; however, these effects were not observed in the tensile and fatigue results.

The measured UTS, YS, and elongation values for the processed IN718-Re alloy at room temperatures—1247 MPa, 909 MPa, and 24%, respectively—align closely with those specified in ASTM F3055-14a. These values are also significantly higher than those specified in the AMS 5383 standard [34] for conventionally processed and heat-treated alloys, which are 962 MPa for UTS, 758 MPa for YS, and 5% for elongation [35]. Additionally, the UTS exceeds the AMS 5663 standard [36] (1236 MPa), while the YS is approximately 9.7% lower (909 MPa vs. 1034 MPa), and the elongation is markedly higher (24% vs. 5%). The tensile properties of the rhenium-modified IN718-Re were slightly superior to those reported by Amato et al. [37], who measured an ultimate tensile strength (UTS) of 1140–1200 MPa, yield strength (YS) of 850–930 MPa, and elongation of 28–30% for PBF-LB/M-processed, unmodified IN718 post-processed by HIP and annealing. In comparison, the UTS and YS values for PBF-LB/M-processed IN718 under similar HIP and heat treatment conditions reached 1391 MPa and 1143 MPa, respectively, with a comparable elongation of 24.9% [26].

At elevated temperatures, the alloy showed a decrease in mechanical properties. The UTS and yield strength (YS) decreased significantly at 650 °C and 760 °C, reflecting the thermal stability limits of the material. Specifically, the tensile strength at 650 °C was approximately 30% lower than at room temperature, a behavior typical for In718 [38]. The UTS and YS values obtained at elevated temperatures are comparable to those of cast In718 subjected to HIP treatment [34]. However, they are lower than those reported for In718 without rhenium addition produced via PBF-LB/M [26]. This reduction is directly attributed to the coarse-grained microstructure observed in the material. Furthermore, at 760 °C, the tensile strength of In718-Re is slightly lower than the values reported for standard In718 in [39]. When considering the decrease in tensile strength at 650 °C and 760 °C, it is essential to note the significant elongation values achieved under these conditions, reaching approximately 15% at 650 °C and 7.5% at 760 °C.

The quasi-cleavage fracture behavior observed at 760 °C further indicates a transition toward brittleness, which can limit the applicability of In718-Re in extremely high-temperature environments. Despite this, the alloy’s performance at 650 °C remains promising for moderately high-temperature aerospace and energy applications.

The measured values of UTS and elongation indicate that the alloying addition did not significantly affect the tensile properties of the alloy at room and elevated temperatures. The high ductility of the IN718-Re alloy, along with its lower-than-average UTS/YS compared to standard IN718, is primarily attributed to its homogenized microstructure. This microstructure is characterized by a large grain size (ASTM grain size 4 or larger), the elimination of the cellular-dendritic structure typically observed in PBF-LB/M In718 in a non-homogenized state, and a high density of twin boundaries. Other potential contributing factors may be the response of the alloy to the precipitation of the γ′ and γ″ phases during heat treatment. The hardness values measured for the as-built, HT, and HIP + HT conditions (301 ± 8, 493 ± 34, and 460 ± 14, respectively) show typical hardness ranges for PBF-ed In718 [9]. The hardness for HIP-ed samples is lower than for HT-ed only, as it is due to the removal of cellular-dendritic structure [40]. However, the 3 wt.% addition of rhenium to IN718 likely contributes to the observed increase in hardness through matrix strengthening. As a result, the relative contribution of γ′ and γ″ phases to hardness may be slightly diminished compared to their typical role in the alloy.

Additionally, a lack of significant hardness improvement aligns with the findings from previous studies on the alloying of rhenium in Inconel 706, confirming similar trends in its effect on alloy properties [41]. In the cited study, the addition of Re to In718 is ineffective in significantly enhancing the mechanical properties or stabilizing the microstructure at elevated temperatures. This is attributed to the high solubility of Re in γ″ precipitates, which reduces supersaturation at interfaces and minimizes its role in hindering precipitate coarsening or grain boundary movement [42]. Therefore, the primary factor contributing to the improved ductility and comparatively lower UTS/YS in post-treated IN718-Re is its homogenized alloy structure, with the weakened γ′ and γ″ phases playing a minor role as well.

Fatigue testing revealed that the fatigue strength of the In718-Re alloy was below 400 MPa for 10^7^ cycles, a value comparable to the fatigue strength typically reported for standard In718 without rhenium addition [43,44]. The critical influence of internal defects, especially near-surface defects, in initiating fatigue cracks was evident across all stress levels. These defects are inherent to the PBF-LB/M process but were not entirely eliminated even though HIP treatment was applied. At higher stress levels (600 MPa), twin boundaries emerged as an additional cause of failure. This dual fatigue failure mechanism highlights the complex interplay between structural features and loading conditions. The persistence of internal defects suggests that further optimization of the PBF-LB/M manufacturing process of In718-Re is required.

The results presented in this study indicate that the rhenium alloying addition did not significantly affect the tensile and fatigue properties of the In718 alloy. In the Ni–Fe superalloys, an increase in the Al/Nb ratio is considered a more effective approach to stabilize the microstructure by promoting γ′ precipitates [41]. However, this study demonstrates a key outcome: surface-modified IN718 powder can be successfully used to achieve complete dissolution of refractory alloying elements, including high-melting-point metals such as rhenium, tungsten, or niobium, in situ during the additive manufacturing process. This provides a novel pathway for incorporating such elements into nickel-based alloys through laser-based powder bed fusion.

Internal defects were among the most significant factors affecting the static and fatigue properties of In718-Re. Modifying the PBF process to reduce internal material discontinuities is a promising direction for further research. Studies on AM processes supported by ultrasound waves [45,46] or electromagnetic fields [47] show that this approach reduces internal defects and refines the deposited material microstructure. It indicates a high potential for enhancing laser-based AM processes by non-contact treatment methods and improving the structural and mechanical properties of the processed materials. Therefore, further research on the additive manufacturing of Re-modified Ni-based alloys should focus on optimizing the parameters of the PBF-LB, HIP, and HT processes to enhance mechanical properties, reduce high-temperature creep, and improve oxidation resistance at elevated temperatures.

The rhenium-reduced high-temperature creep of SX Ni-based alloys, such as CMSX-4, CMSX-10, and Rene N6 [41,48], provides a solid foundation for continued work in this area.

## 5. Conclusions

This study demonstrates that rhenium alloying of Inconel 718, combined with post-processing treatments (HIP + HT), leads to average tensile strength (near ASTM standards minimums), high ductility, and average fatigue performance. Rhenium addition has been found to be an ineffective approach for improving the mechanical properties or stabilizing the microstructure during service at elevated temperatures. The main conclusions are:

1. The innovative powder surface modification method (Inconel 718 powder alloyed with 3 wt.% rhenium) ensured homogeneous rhenium distribution, effectively mitigating additive segregation and unmelted particle defects commonly observed in powders alloyed via conventional blending.
2. The addition of rhenium has been found ineffective for enhancing the mechanical properties or stabilizing the microstructure during service at elevated temperatures. The performance of the alloy decreased significantly above 650 °C, highlighting its limitations for high-temperature applications.
3. The results demonstrate that the mechanical properties are primarily governed by the high-temperature post-treatment process, which facilitated a homogenized microstructure and large grain size.
4. Near-surface defects and twin boundaries were identified as critical crack initiation sites, emphasizing the necessity for further optimization of PBF-LB/M processing parameters to improve fatigue performance.

While rhenium addition shows limited benefits in Inconel 718, the study underscores the potential of in situ alloying through powder surface modification as a flexible method for incorporating high-melting-point elements into nickel-based alloys for tailored alloy design in additive manufacturing.

## Figures and Tables

**Table 1:** Post-treatment processes, standards for geometry and finishing (machining), and tests performed for the series of In718-Re specimens.

Specimen Group	Post-Treatment	Sample Geometry /Finishing Standard	Test
HIP + HT/@RT	HIP + solution annealing + double aging	ASTM E8/E8M-22	static tensile test, room temp.
HIP + HT/@650			static tensile test, 650 °C (1200 F)
HIP + HT/@760			static tensile test, 760 °C (1400 F)
HIP + HT		ASTM E466	fatigue test, room temp.

## Data Availability

The raw data supporting the conclusions of this article will be made available by the authors on request.
